# A Novel Cell Line Derived from Pleomorphic Adenoma Expresses MMP2, MMP9, TIMP1, TIMP2, and Shows Numeric Chromosomal Anomalies

**DOI:** 10.1371/journal.pone.0105231

**Published:** 2014-08-19

**Authors:** Aline Semblano Carreira Falcão, Maria Sueli da Silva Kataoka, Nélson Antonio Bailão Ribeiro, José Antonio Picanço Diniz, Sérgio Melo Alves, André L. Ribeiro Ribeiro, Adriane Sousa de Siqueira, Artur Luiz da Silva, Rommel Thiago Jucá Ramos, Vanessa M. Freitas, Ruy G. Jaeger, João J. V. Pinheiro

**Affiliations:** 1 Department of Oral and Maxillofacial Pathology, School of Dentistry, Federal University of Pará-UFPA, Belém, PA, Brazil; 2 Laboratory of Molecular Biology, Evandro Chagas Institute, Belém, PA, Brazil; 3 Laboratory of Electron Microscopy, Evandro Chagas Institute, Belém, PA, Brazil; 4 Department of Oral and Maxillofacial Surgery, School of Dentistry, Universitary Center of Pará-CESUPA, Belém, Pará, Brazil; 5 Department of Cell and Developmental Biology, Institute of Biomedical Sciences, University of São Paulo, São Paulo, Brazil; 6 Institute of Biological Sciences, Federal University of Pará (Universidade Federal do Pará—UFPA), Belém, Pará, Brazil; Virginia Commonwealth University, United States of America

## Abstract

Pleomorphic adenoma is the most common salivary gland neoplasm, and it can be locally invasive, despite its slow growth. This study aimed to establish a novel cell line (AP-1) derived from a human pleomorphic adenoma sample to better understand local invasiveness of this tumor. AP-1 cell line was characterized by cell growth analysis, expression of epithelial and myoepithelial markers by immunofluorescence, electron microscopy, 3D cell culture assays, cytogenetic features and transcriptomic study. Expression of matrix metalloproteinases (MMPs) and their tissue inhibitors (TIMPs) was also analyzed by immunofluorescence and zymography. Furthermore, epithelial and myoepithelial markers, MMPs and TIMPs were studied in the tumor that originated the cell line. AP-1 cells showed neoplastic epithelial and myoepithelial markers, such as cytokeratins, vimentin, S100 protein and smooth-muscle actin. These molecules were also found *in vivo*, in the tumor that originated the cell line. MMPs and TIMPs were observed *in vivo* and in AP-1 cells. Growth curve showed that AP-1 exhibited a doubling time of 3.342 days. AP-1 cells grown inside Matrigel recapitulated tumor architecture. Different numerical and structural chromosomal anomalies were visualized in cytogenetic analysis. Transcriptomic analysis addressed expression of 7 target genes (VIM, TIMP2, MMP2, MMP9, TIMP1, ACTA2 e PLAG1). Results were compared to transcriptomic profile of non-neoplastic salivary gland cells (HSG). Only MMP9 was not expressed in both libraries, and VIM was expressed solely in AP-1 library. The major difference regarding gene expression level between AP-1 and HSG samples occurred for MMP2. This gene was 184 times more expressed in AP-1 cells. Our findings suggest that AP-1 cell line could be a useful model for further studies on pleomorphic adenoma biology.

## Introduction

Pleomorphic adenoma is the most frequent salivary gland benign neoplasm, and largely affects parotid glands (80% of cases), with a discrete female predominance. Pleomorphic adenoma consists of an epithelial and myoepithelial cells mixture embedded in a mesenchyma-like stroma [1], [2]. This tumor usually presents a benign behavior, but can recur after inappropriate treatment [3]. Furthermore, about 2–8.5% of cases may undergo malignant transformation [4], [5].

Despite its slow growth, pleomorphic adenoma can be locally invasive and, whether not treated promptly, may produce significant morbidity [6]. Since this benign neoplasm shows low mitotic index [7], cell proliferation rate does not seem to be directly related to its invasiveness.

Pleomorphic adenoma exhibits a prominent extracellular matrix (ECM), which regulates tumor growth and progression [8], [9]. ECM molecules are modified by matrix metalloproteinases (MMPs), a family of enzymes that can modulate cell fate by creating space for migration, releasing ECM-bound growth factors and activating signaling molecules [10]–[12].

MMPs play important roles during aggressive tumors development, since invasiveness of neoplastic cells has been associated with overexpression of MMPs and altered expression of their tissue inhibitors (TIMPs) [9], [13]. Among different proteases, MMP2 and MMP9 are key regulators of cancer [14], [15]. Thus, the balance between these enzymes and their inhibitors are crucial to determine tumor invasiveness.

The underlying recurrence and malignant change mechanisms of salivary gland pleomorphic adenoma are still not clear, and intrinsic biological factors such as MMP-TIMP system might have an important part [13]. However, to our knowledge, no studies have attempted to address the machinery that regulates remodeling and local invasiveness of this tumor.

Protein expression is directly related to genetic control [16]. More than half of solid tumors show numeric and/or structural chromosomal abnormalities. Chromosomal rearrangements can be directly involved in tumorigenesis and affect pro-oncogenes, tumor suppressor genes and cell cycle-related cell genes [17]. Therefore, cytogenetic analysis is important not only for tumor diagnosis and prognosis, but also to improve our understanding of a neoplasm behavior.

*In vitro* systems have been used to study tumor biology. Regarding pleomorphic adenoma, only a few cells lines have been established [18]–[21]. Kondo *et al.* [18] established a cell line (Nagoya-78) from a benign pleomorphic adenoma of the lip, with further analysis of chromosomal abnormalities. Jaeger *et al.* [19] established a cell line (AP2) from a pleomorphic adenoma of the parotid gland, which showed myoepithelial-like characteristics in a three dimensional culture. Kitagawa et al. [20] established immortalized human pleomorphic adenoma cells by infecting them with a retroviral vector containing hTERT. Maruyama et al. [21] established five cell lines from pleomorphic adenomas of the parotid gland, showing chromosome abnormalities such as ploidies and various kinds of structural abnormalities.

In this study, we established a novel cell line derived from human pleomorphic adenoma to better understand the mechanisms regulating remodeling and local invasiveness of this tumor. Expression of phenotypic markers, MMPs and TIMPs were evaluated by morphological, immunohistochemistry and molecular biology methods. This cell line can be a useful model for further studies of pleomorphic adenoma features.

## Material and Methods

### Ethics Statement

The Ethics Committee of the Institute of Health Sciences, Federal University of Pará approved our study (protocol n° 209/08). The consent form was obtained through a written document explaining in detail the procedures to be carried out such as how to obtain the samples and establish the cell line. In addition, consent for publication of clinical details was provided by the patient from whom we obtained the sample used in the study.

### Collection of pleomorphic adenoma sample

The pleomorphic adenoma sample was obtained from a 35-year-old male, who sought for dental care at University Hospital João de Barros Barreto (Federal University of Pará), reporting an increase in volume in the left maxillary tuberosity. During clinical examination, a sessile and fibrous lesion with nearly 9 cm in diameter was detected, extending from the palate to the median raphe. Incisional biopsy was carried out, and histopathological examination established the diagnosis of pleomorphic adenoma. Portions of the specimen were sent both to immunohistochemical analysis and to establishment of cell cultures.

### Immunohistochemistry

Sections of 3 µm were obtained from a formalin-fixed paraffin-embedded sample of pleomorphic adenoma, and mounted on 3-aminopropyltriethoxysilane-coated slides (Sigma Chemical Co., St. Louis, MO, USA). The immunohistochemistry staining was performed using the streptavidin biotin method with EnVision-HRP (Dako Corporation, Carpinteria, CA, USA), as described elsewhere [11]. Breast carcinoma samples were used as positive control, and non-immune sera served as negative controls. Antibodies against vimentin, α-smooth muscle actin, S-100 and cytokeratins (CK) AE1/AE3, 14 and 19 (Dako), MMP2, MMP9, TIMP1 and TIMP2 (DBS-Diagnostic Biosystem Inc., Pleasanton, CA, USA) were used. All primary antibodies were diluted 1∶200.

### Primary cell culture

A primary cell culture derived from pleomorphic adenoma was established. Briefly, neoplasm fragments were chemically digested with trypsin and then mechanically dissociated with a Pasteur pipette. Cells were placed in 25 cm^2^ culture flasks with culture medium 199 (Gibco-BRL, Grand Island, NY, USA) supplemented with 10% fetal bovine serum (Gibco), and incubated in a humidified atmosphere of 5% CO_2_ at 37°C. AP-1 cell line was derived from a subculture of the primary cell culture.

### Cell growth analysis

AP-1 cells were seeded at a density of 10^4^ cells/ml in 24-well plates in triplicates. Cells were harvested and counted using trypan blue dye exclusion method to assess viability. Cells were counted every 24 hours for 5 days.

### Electron microscopy

For scanning electron microscopy (SEM), AP-1 cells grown on coverslips for 48 hours were fixed by immersion in a solution containing 4% paraformaldehyde and 2.5% glutaraldehyde in sodium cacodylate buffer 0.1 M, pH 7.2, for 1 hour at room temperature. Cells were then post-fixed with 1% osmium tetroxide (OsO4, EMS - Electron Microscopy Sciences, Hatfield, PA, USA) in cacodylate buffer for 15 minutes at 4°C. Samples were dehydrated in graded ethanol and dried using the critical point method (K-850, Elmitech Ltd, Ashford, Kent, UK). Coverslips were mounted on aluminum stubs (1/8" pin diameter - Ted Pella, Redding, CA, USA) with double-sided carbon tape (8 mm, Ted Pella), and sputter-coated with 20 nm gold (K-550, Elmitech). Samples were analyzed using a scanning electron microscope (1450VP, LEO Co. LTD., Cambridge, UK).

For transmission electron microscopy (TEM), cells grown in 75 cm^2^ flasks were fixed in a solution containing 4% paraformaldehyde, 2.5% glutaraldehyde and 5 mM calcium chloride in sodium cacodylate buffer 0.1 M (pH 7.2) at room temperature, and then collected as cell pellets, and post-fixed with a solution containing 1% osmium tetroxide, 0.8% potassium ferrocyanide, and 5 mM calcium chloride in cacodylate buffer. Next, cells were dehydrated in graded acetone, embedded in epoxy resin (Epon 812, EMS) and cured at 70°C for 48 hours. Ultrathin sections were cut and double-stained with uranyl acetate and lead citrate [22]. Specimens were examined in a transmission electron microscope (EM 900, Carl Zeiss).

### 3D cell culture assays

For three-dimensional cultures, AP-1 cells (2×10^4^) were embedded within reduced growth factor Matrigel (BD Biosciences, San Jose, CA) and seeded on Matrigel-pre-coated coverslips. After Matrigel finish jellification at 37°C for 30 minutes, complete medium was added and cells were grown in a 5% CO_2_ humidified incubator at 37°C for 10 days. Samples were fixed in 4% paraformaldehyde, permeabilized using 0.5% Triton X-100 (Sigma) in PBS, and labeled to actin by the specific probe Alexa Fluor 488 Phalloidin (Invitrogen, Carlsbad, CA, USA). Actin staining detected cortical cytoskeleton, thus outlining cell shape. Samples were mounted using Pro Long with DAPI (Invitrogen), and analyzed by confocal microscopy (Carl Zeiss LSM 780-NLO, CEFAP-ICB-USP). Three-dimensional reconstructions were carried out by Volocity software (PerkinElmer, Waltham, MA, USA).

### Immunofluorescence

AP-1 cells were fixed in 2% paraformaldehyde for 10 minutes, and permeabilized with 0.5% Triton X-100 (Sigma) in PBS for 5 minutes. Samples were blocked using 10% goat serum, and incubated with primary antibodies against vimentin, α-smooth muscle actin, S-100 (dilution 1∶100, Dako), CK-AE1/AE3, CK-14 and CK-19 (1∶50 dilution, Dako), MMP2, MMP9, TIMP1 and TIMP2 (1∶50 dilution, DBS). All primary antibodies were incubated for 18 hours at 4°C. Solutions containing secondary antibodies conjugated to FITC (fluorescein isothiocyanate - ICN Biomedicals Inc., Costa Mesa, CA, USA) or TRITC (tetramethyl rhodamine isothiocyanate - Zymed, San Francisco, CA, USA) were applied at room temperature for 1 hour. Hoechst 33258 (1∶1000 dilution, Sigma) was used for nuclear counterstaining. Non-immune sera served as negative controls. Samples analysis and image acquisitions were performed using Axioscope A1 fluorescence microscope equipped with AxioCam MRC digital camera (Carl Zeiss, Oberköchen, Germany).

### Protease Activity

Complete growth medium was replaced by serum-free medium for 24 hours in AP-1 cells cultured on a six-well plate. Conditioned medium was collected, treated with protease inhibitors (pepstatin A, PMSF and E-64, Santa Cruz Biotechnology Inc., Paso Robles, CA, USA), and subjected to zymography. Briefly, conditioned medium was concentrated (Microcon 30 K, Millipore Co, Bedford, MA, USA) and resuspended in SDS-PAGE sample buffer (without β-mercaptoethanol). Protein amount was estimated by BCA assay (Pierce, Rockforf, IL, USA). Samples were separated on 10% polyacrylamide gels containing 0.2% gelatin (Sigma). After electrophoresis, gels were washed in 2.5% Triton X-100 (Sigma) for 30 min and incubated for 16–24 hours at 37°C in a development buffer containing 50 mM Tris pH 8.0, 5 mM CaCl_2_ and 0.02% NaN_3_. Gels were stained with 0.2% Coomassie blue R250 (Amersham Co Arlington Heights, IL, USA) and destained with acetic acid/methanol. MMP2 and MMP9 activity was observed as clear bands in dark background.

### Cytogenetic study

Chromosomes were obtained by adding 0.0016% colchicine solution (10 IU) to each culture flask of AP-1 cells for 1 hour. Hypotonization of samples with 0.056% KCl, fixation with Carnoy and preparation of slides were performed as described elsewhere [23], followed by staining procedure for G banding [24]. Slides were analyzed using Axiophot microscope (Carl Zeiss), and image acquisition was carried out using the Case Data Manager software (version 6.0, Applied Spectral Imaging, Vista, CA, USA). Karyotypes were mounted based on the morphology and decreasing size order using the View Banda software (version 5.5, Applied Spectral Imaging). Karyotypes were visually compared with standards karyotypes presented by Thompson et al. [25].

### Transcriptomic study

#### Data evaluation and processing

Raw data quality, obtained through the Ion Torrent PGM sequencing using two chips (316), was evaluated by FastQC [26]. This process allowed us to identify the reads regions with low quality bases and also the length distribution of the reads, which were used to process the data. The reads obtained through AP-1 and HSG (normal salivary gland cell line) libraries sequencing were processed by Fastx (http://hannonlab.cshl.edu/fastx_toolki/), in order to remove the low quality ends of the sequences (bases with quality bellow Phred 20), and adapters represented by the first four bases of the read beyond remove the reads smaller than 50 bp.

#### Gene Expression

The reads of AP-1 and HSG libraries were mapped with Human reference genome (GRCh37 - available at http://www.ensembl.org), using the Torrent Mapping Alignment Program for Ion Torrent – TMAP (http://mendel.iontorrent.com/ion-docs/Technical-Note-TMAP-Alignment_9012907.html), and selecting the option *mapall* for the mapping and the parameter –max-seed-band (the window of bases in which the group seeds) with the value 18. TMAP was performed with four algorithms simultaneously: BWA-short [27], BWA-long [28], SSAHA long-read algorithm [29] and Super-maximal Exact Matching [30]. The Samtools [31] converted the SAM files (produced by the libraries mapping into reference genome) to the BAM format (used to measure the gene expression level), using the last part of the Cufflinks Differencial Expression pipeline [32].

## Results

### Histopathology

**Figure 1:**
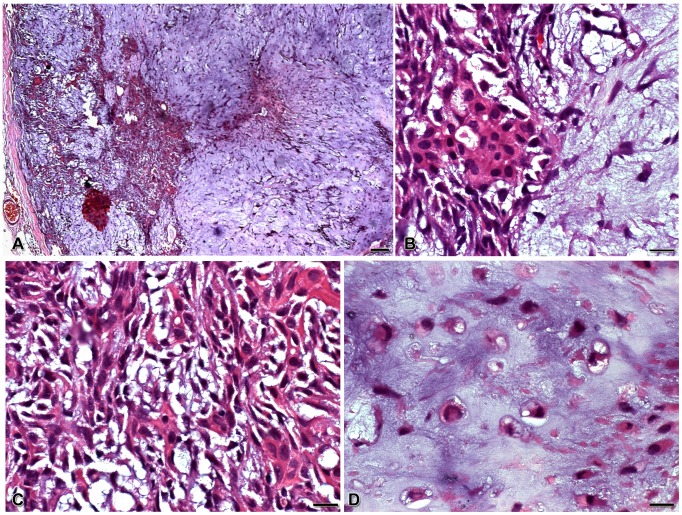
Histopathological features of pleomorphic adenoma sample stained with hematoxilin & eosin. Samples are tissue sections from the patient from whom the AP-1 cell line was derived. A fibrous tissue capsule surrounds a heterogeneous and dense epithelial cell population, distributed as sheets, cords and islets (**A**). Tumor cells show spindle or plasmacytoid phenotype (**B**), and are embedded in myxoid (**C**) and chondroid (**D**) stroma. Scale bars: A = 50 µm; B, C, D = 10 µm.

The tumor that originated the AP-1 cell line exhibited typical histopathological features of pleomorphic adenoma, with presence of a fibrous capsule surrounding a dense population of epithelial cells, distributed as sheets, cords and islets (**Fig. 1A**). These cells had mainly a spindle or plasmacytoid aspect (**Fig. 1B**), and were embedded in a myxoid or chondroid stroma (**Fig. 1C, D**).

### Immunohistochemistry

**Figure 2:**
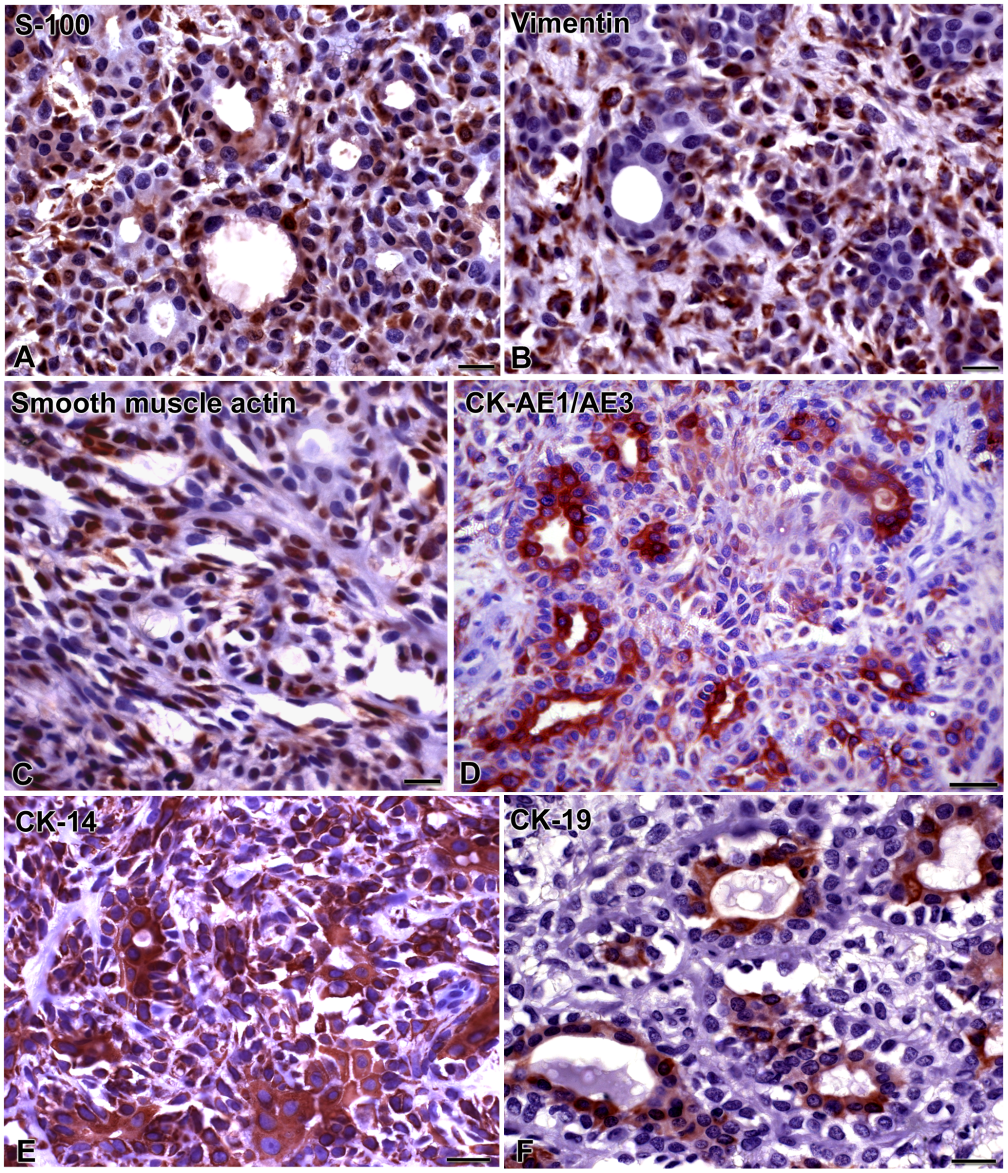
Pleomorphic adenoma expresses epithelial and myoepithelial markers *in vivo*. Samples are tissue sections from the patient from whom the AP-1 cell line was derived. S-100 protein (**A**) exhibits cytoplasmic labeling, mostly in plasmacytoid cells. Cytoplasmic expression of vimentin (**B**) and smooth muscle actin (**C**) is observed in spindle and plasmacytoid cells. Cytokeratins AE1/AE3 (**D**) and CK-14 (**E**) show cytoplasmic staining in ductal, plasmacytoid and spindle cells. CK-19 (**F**) expression is found in ductal cells. Scale bars: A, B, C = 10 µm; D, E, F = 20 µm.

Immunohistochemistry of the pleomorphic adenoma that originated AP-1 cells showed that S-100 protein expression was observed especially in plasmacytoid cells (**Fig. 2A**). Vimentin (**Fig. 2B**) and smooth muscle actin (**Fig. 2C**) were observed in spindle and plasmacytoid cells. CK-AE1/AE3 (**Fig. 2D**) and CK-14 (**Fig. 2E**) showed cytoplasmic expression in ductal, spindle and plasmacytoid cells. On the other hand, CK-19 (**Fig. 2F**) was detected predominantly in ductal cells.

**Figure 3:**
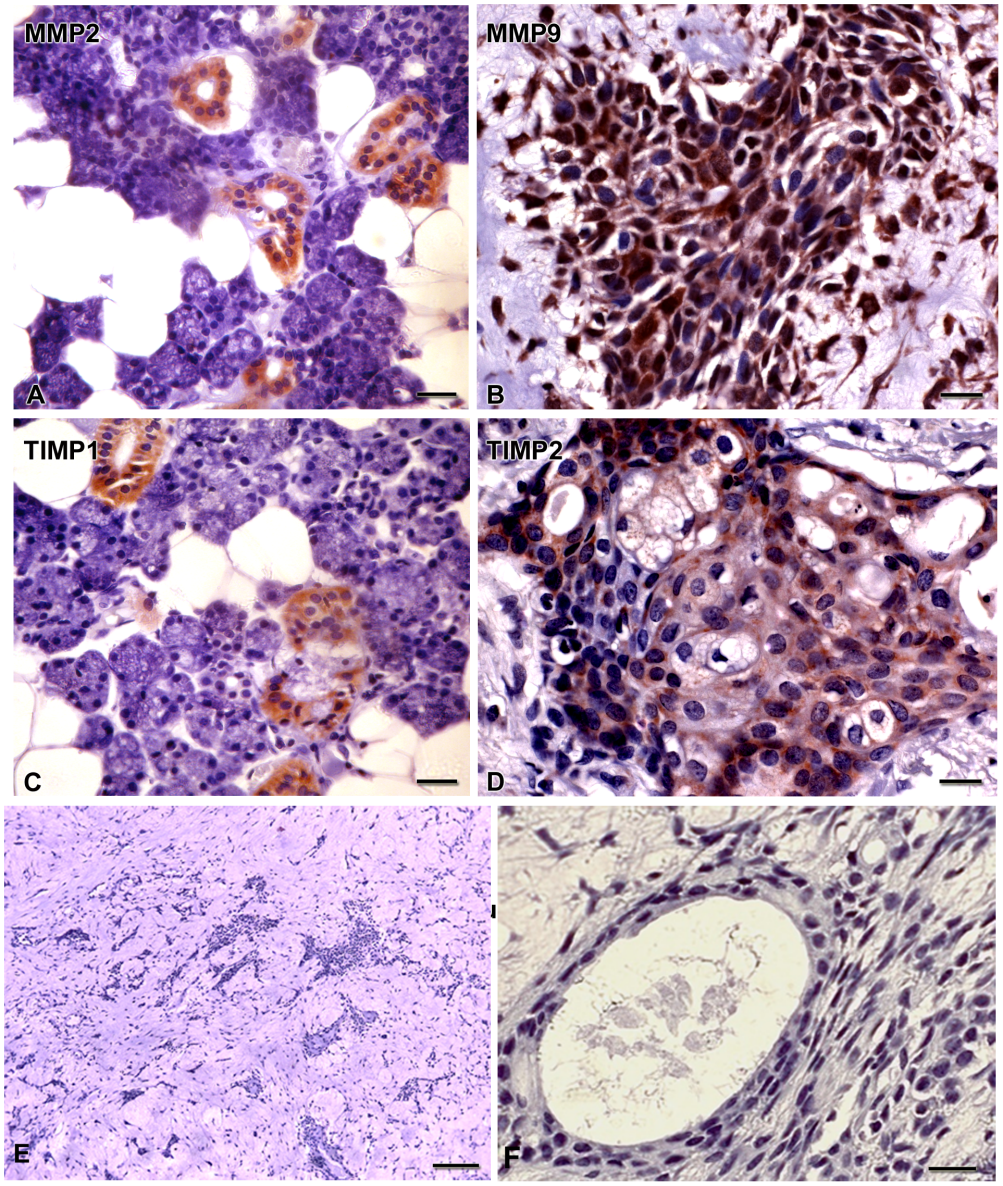
Pleomorphic adenoma expresses MMPs and TIMPs *in vivo*. Samples are tissue sections from the patient from whom the AP-1 cell line was derived. MMP2 (**A**) is expressed in ductal cells, while MMP9 (**B**) is observed in spindle and plasmacitoyd cells. TIMP-1 (**C**) is observed in luminal cells of duct-like structures, and TIMP-2 (**D**) is found in spindle and plasmacytoid cells. Negative controls show no staining (E, F). Scale bars: 10 µm; E, F = 20 µm.

MMP2 (**Fig. 3A**) was detected in ductal cells and MMP9 (**Fig. 3B**) was observed in spindle and plasmacitoyd cells. Luminal cells of duct-like structures showed TIMP1 expression (**Fig. 3C**). TIMP2 (**Fig. 3D**) was found in spindle and plasmacytoid cells. Positive controls of all antibodies exhibited cytoplasmic labeling of breast carcinoma cells (not illustrated), and negative controls showed no staining (**Fig. 3** **E, F**).

### Cell culture and growth analysis

**Figure 4:**
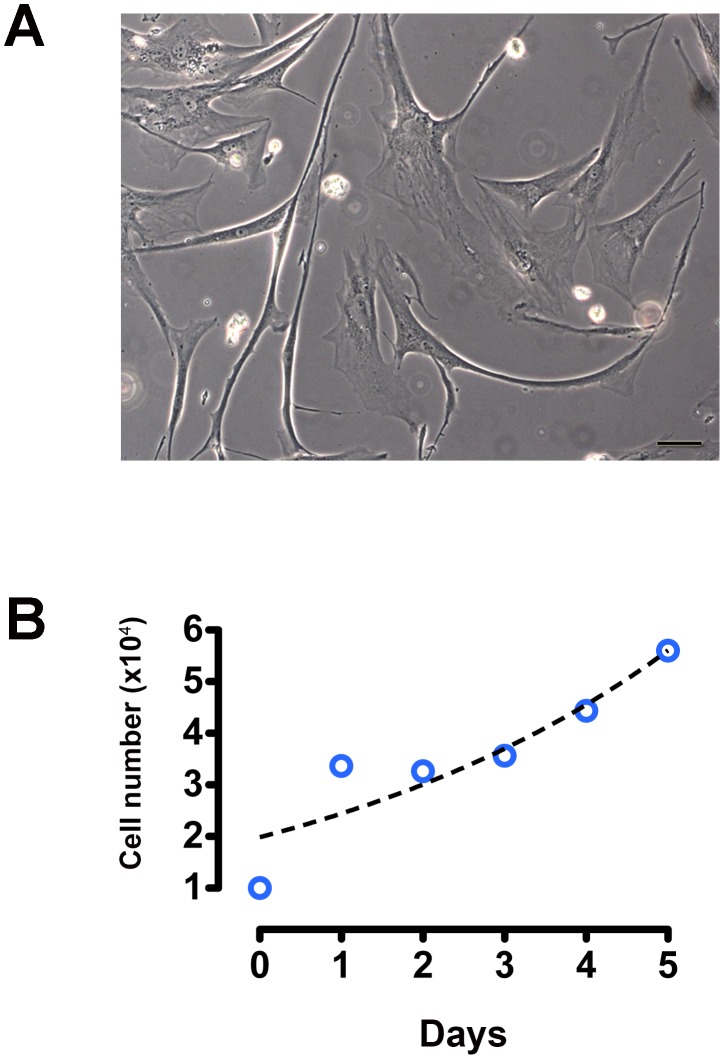
Cell culture and growth analysis. Phase contrast microscopy displays AP-1 cells with polyhedral and spindle-shaped phenotype (**A**). Growth curve of AP-1 cells shows growth until the 5^th^ day (**B**). Doubling time was 3.342 days. Points were fitted using exponential growth non-linear regression. Scale bar: A = 20 µm.

In culture, AP-1 cells showed polyhedral and spindle-shaped phenotype (**Fig. 4A**). Growth curve showed that AP-1 cells grew steadily until the 5^th^ day, with a doubling time of 3.342 days (**Fig. 4B**).

### Electron microscopy

**Figure 5:**
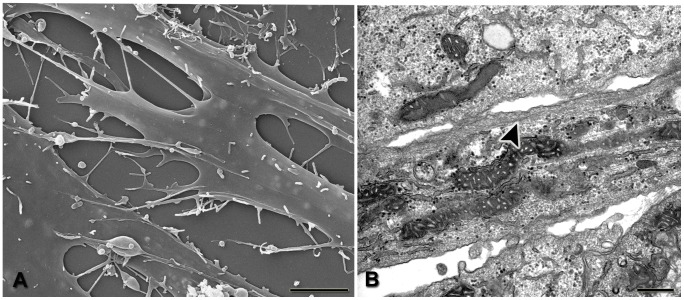
Characterization of AP-1 cells. SEM shows cells with elongated and flattened morphology, with numerous thin cytoplasmic processes forming an intricate network (**A**). Analysis by TEM reveals the presence of well-developed Golgi complex, mitochondria, and large amounts of glycogen granules. Filamentous networks are found in the cytoplasm near the plasma membrane (**B, arrowhead**). Scale bars: A = 5 µm; B = 500 nm.

AP-1 cells, when observed through SEM, exhibited elongated and flattened morphology with numerous and thin cytoplasmic processes adhered to substrate and forming an intricate network. Delicate filopodia were found protruding from the cell surface (**Fig. 5A**). Ultra structural analysis by TEM revealed the presence of well-developed Golgi complex and mitochondria, large amounts of glycogen granules, and networks of filamentous structures in the cytoplasm near the plasma membrane (**Fig. 5B** **, arrowhead**).

### 3D cell culture assay

**Figure 6:**
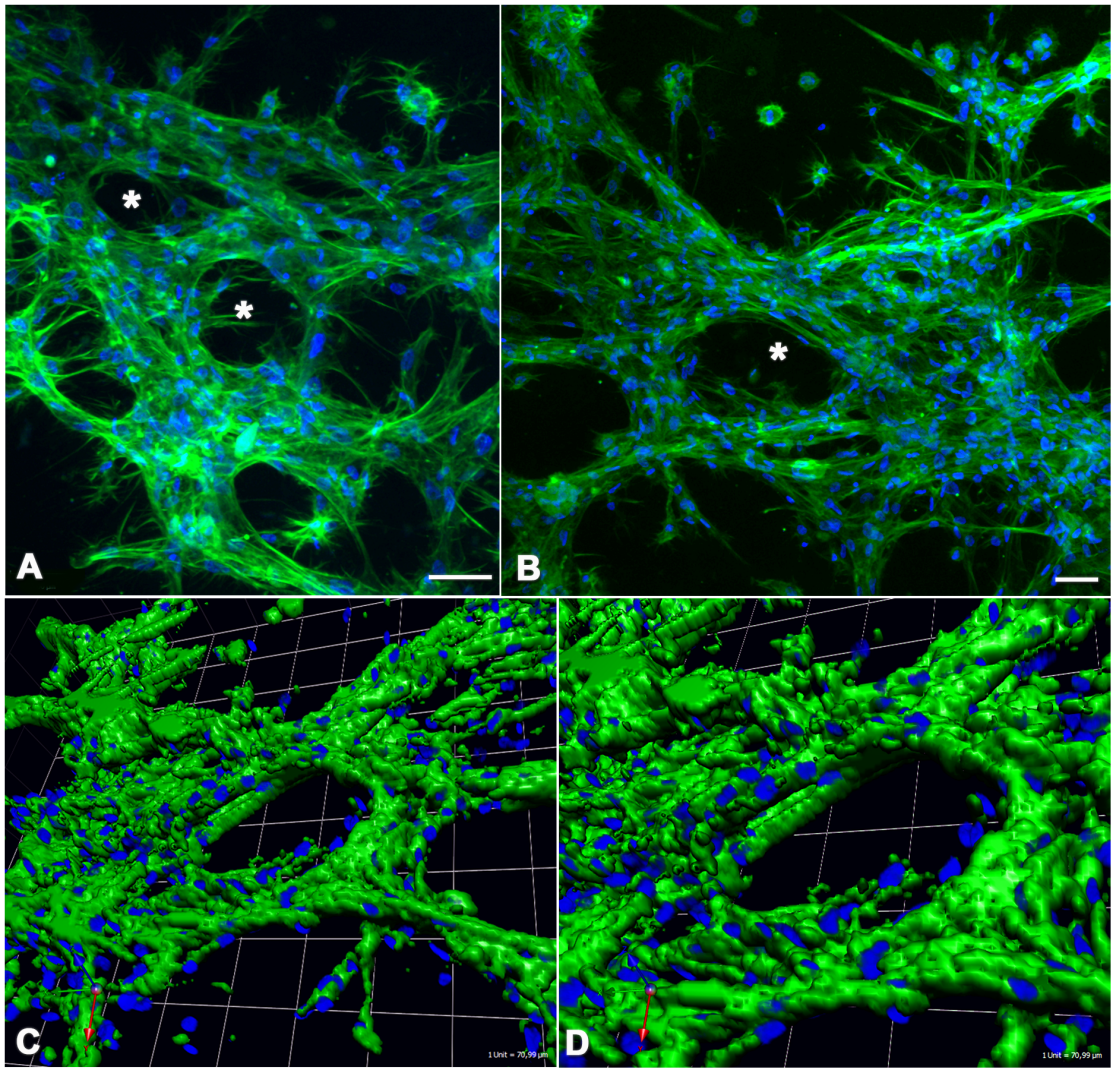
3D cell culture assays. AP-1 cells were grown within Matrigel, fixed and labeled with actin and DAPI. Actin staining reveals the cortical cytoskeleton, thus outlining cell boundaries. AP-1 cells grown inside Matrigel appear epitheliod, forming closely packed arrangements (**A and B**). Duct-like structures are observed (**A and B, asterisks**). Three-dimensional reconstruction by Volocity software clearly shows these duct-like spaces formed by multiple layers of cells (**C and D**). Overall, these features recapitulate pleomorphic adenoma *in vivo* phenotype. Scale bar: 50 µm.

Actin-labeled AP-1 cells grown inside Matrigel appeared epithelioid, forming closely packed arrangements (**Figs. 6A and B**). Duct-like structures were observed (**asterisks in** **Figs. 6A and B**). Three-dimensional reconstruction by Volocity software clearly shows these duct-like spaces formed by multiple layers of cells (**Figs. 6C and D**). Overall, these features recapitulate pleomorphic adenoma *in vivo* phenotype.

### AP-1 cells express myoepithelial markers, MMPs and TIMPs

**Figure 7:**
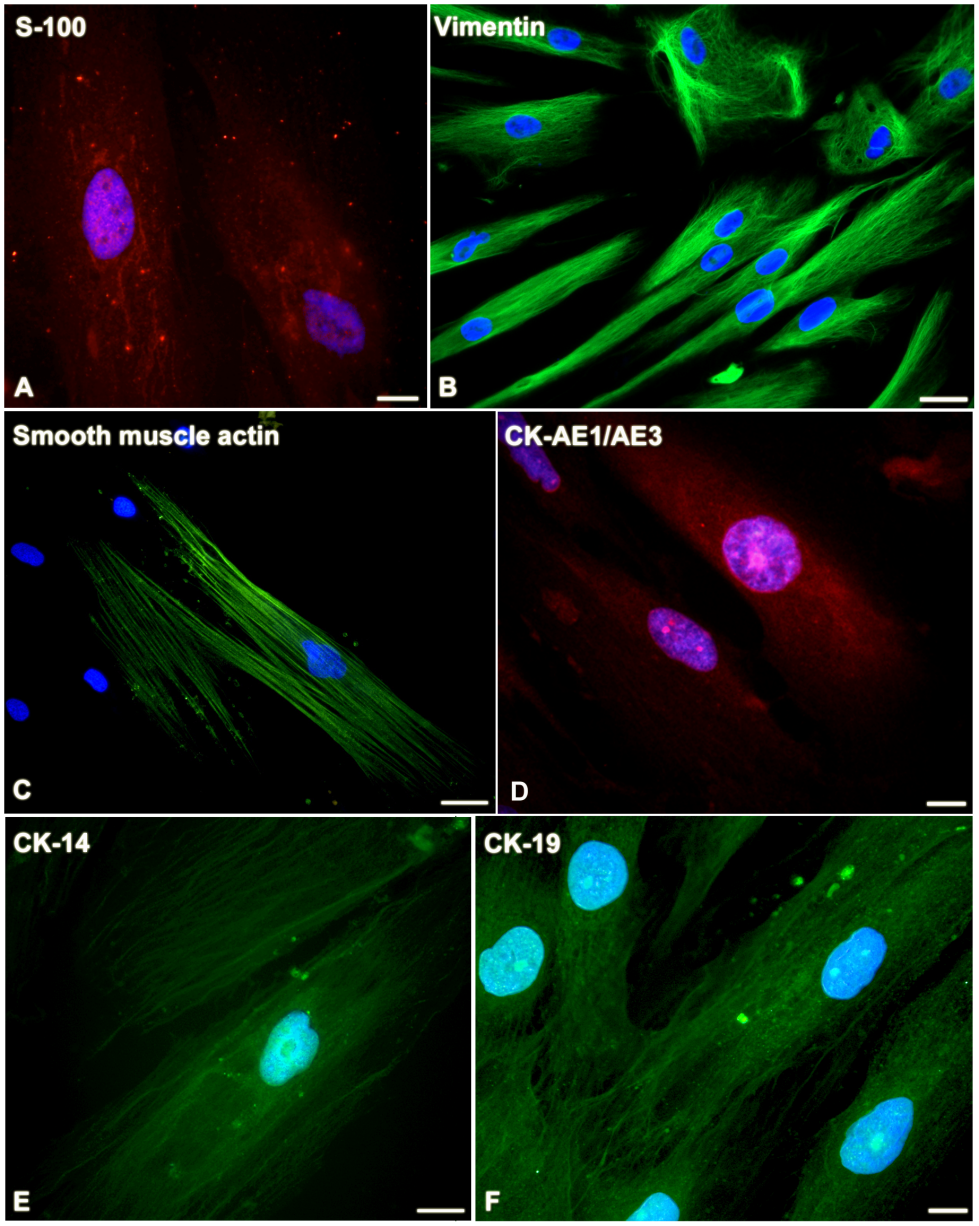
Pleomorphic adenoma expresses epithelial and myoepithelial markers *in vitro*. S-100 (**A**) is observed as dots distributed throughout cell cytoplasm. Vimentin (**B**), smooth muscle actin (**C**), CK-AE1/AE3 (**D**), CK-14 (**E**) and CK-19 (**F**) are present as filamentous network. Scale bars: A, D, F = 10 µm; B, C, E = 20 µm.

Expression of S-100 (**Fig. 7A**) was identified as dots distributed throughout cell cytoplasm. Vimentin (**Fig. 7B**), smooth muscle actin (**Fig. 7C**), CK-AE1/AE3 (**Fig. 7D**), CK-14 (**Fig. 7E**) and CK-19 (**Fig. 7F**) were exhibited as filamentous staining.

**Figure 8:**
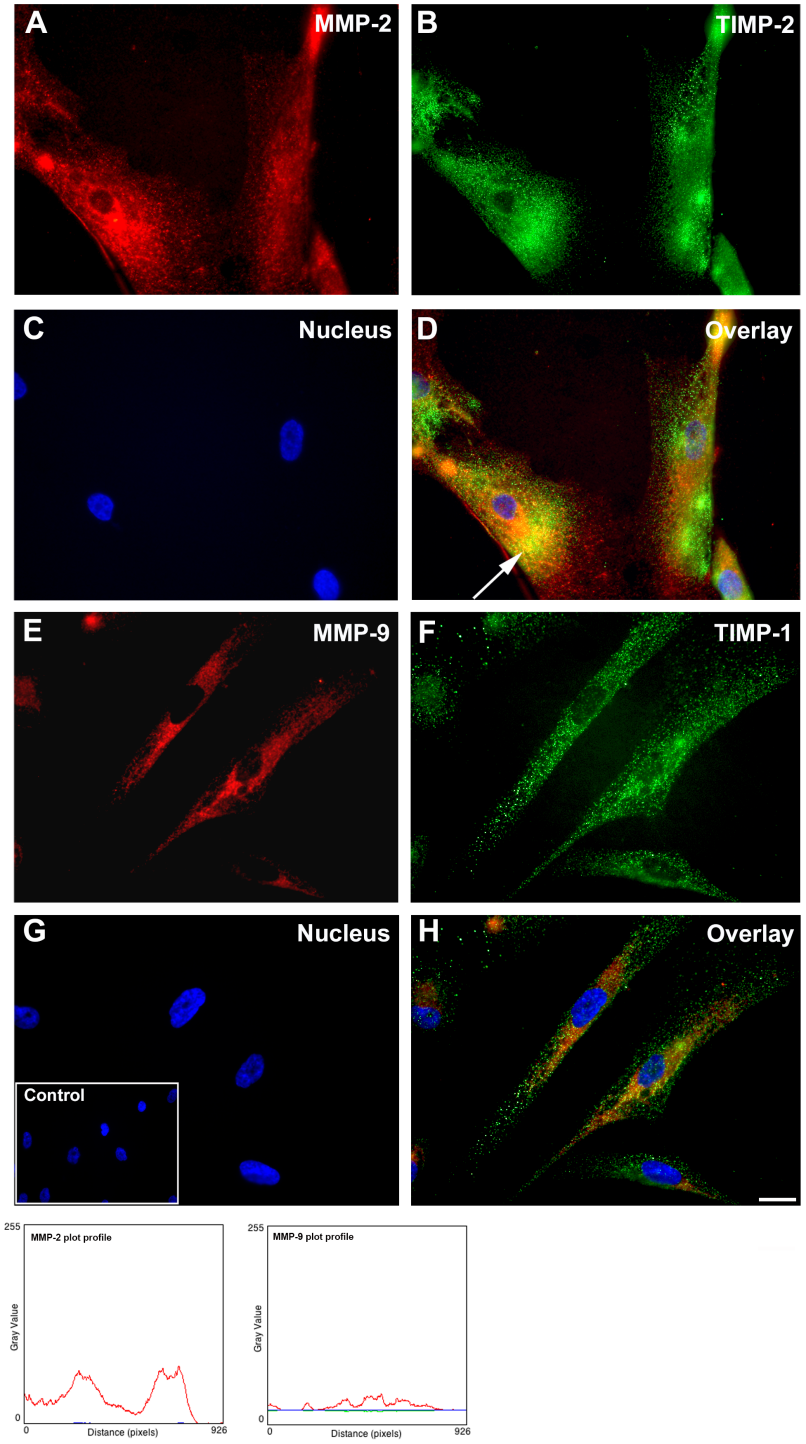
MMPs and TIMPs colocalize *in vitro*. AP-1 cells exhibit MMP2/TIMP-2 (**A, B, D**) and MMP9/TIMP-1 (**E, F, H**) as colocalized dots (**D, arrow**). Quantitative fluorescence shows that MMP2 staining was clearly higher compared to MMP9 (**bottom plots**). Negative control exhibits no staining (**boxed area**). Scale bar: 20 µm.

MMPs (**Figs. 8** **A, E**) and TIMPs (**Fig. 8** **B, F**) showed similar patterns of localization exhibiting punctate staining throughout the cytoplasm. Furthermore, these proteins were co-localized (**Figs. 8** **D, H**). Quantitative fluorescence showed that MMP2 staining was clearly higher compared to MMP9 (**Fig. 8** **, bottom plots**). Negative controls showed no staining (**Fig. 8** **, boxed area**).

### AP-1 cells exhibit protease activity

**Figure 9:**
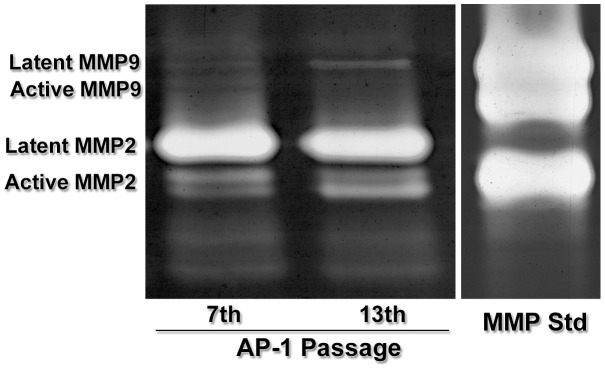
Protease activity. Conditioned media of AP-1 cells in the 7th and 13th passages were analyzed by zymography. Gelatinolytic bands corresponding to MMP2 and MMP9 are observed in both groups. Both active and latent forms of MMP2 are observed. MMP9 latent form seems to be secreted by cells in the 13^th^ passage. MMP2 and MMP9 positive controls (Std MMP) are included. Zymographic experiments were carried out at least three times with consistent results.

In zymograms, conditioned media of AP-1 cells in the 7^th^ and 13^th^ passages induced formation of gelatinolytic bands corresponding to molecular weights of MMP2 and MMP9 (**Fig. 9**). AP-1 cells secreted both active and latent forms of MMP2. MMP9 latent form appeared to be more evident in samples from the 13^th^ passage. MMPs positive controls (**Fig. 9** **, Std MMP**) were analyzed in the same gel to confirm the results.

### Cytogenetic studies

**Figure 10:**
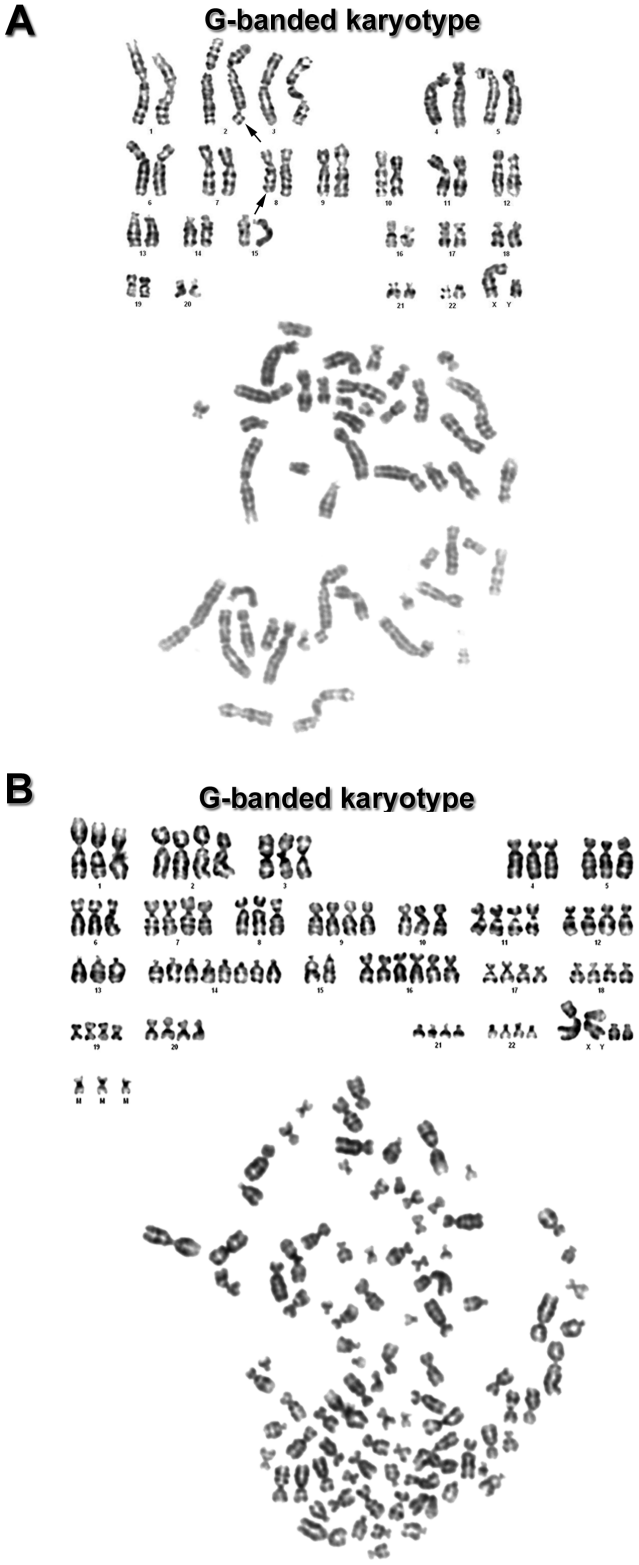
Numerical and structural chromosomal abnormalities. G-banded karyotype, euploid metaphases (**A**) shows rearrangements of terminal portions of chromosomes 2 and 8 long arms. Polyploid metaphases (**B**) reveal numerical variations for different chromosomes.

A total of 37 metaphases of 7^th^ AP-1 passage were examined, and the number of chromosomes found in metaphase dispersions ranged from 46 to 92. The rate of euploid/polyploid metaphases was 15/22, meaning that about 60% of metaphases were polyploid. G-banding analysis in euploid metaphases showed terminal portions rearrangements on the long arm of chromosomes, with higher frequency in chromosome 8, and with lower frequency in chromosomes 2, 4, 6 and 16 (**Fig. 10A**). G-banded polyploid cells demonstrated numerical variations for different chromosomes (**Fig. 10B** **and** **Table 1**). Among numerical abnormalities, tetrasomy and trisomy were largely found. Other numerical abnormalities were identified, but at lower frequency, as hexassomy, heptassomy, octassomy, supernumerary chromosomes and micro chromosomes (**Table 1**).

**Table 1:** Numerical chromosomal abnormalities from AP-1 cell line in the 7^th^ passage.

Group	A			B		C & X								D			E			F		G		
N° chromosome	1	2	3	4	5	6	X	7	8	9	10	11	12	13	14	15	16	17	18	19	20	21	22	Y
**Nulisomy**																								
**Monosomy**							3			1														3
**Disomy**		1				2	4		1	1	1	1				1								4
**Trisomy**	3	1	4	2	3	3		2	2		2		1	3		1	3				2	1	1	
**Tetrasomy**	4	5	3	4	4	2		4	2	3	1	6	5	4	6	3	3	7	6	7	4	5	5	
**Pentasomy**				1				1	1	1	1				1	1					1	1	1	

### Transcriptomic study

**Figure 11:**
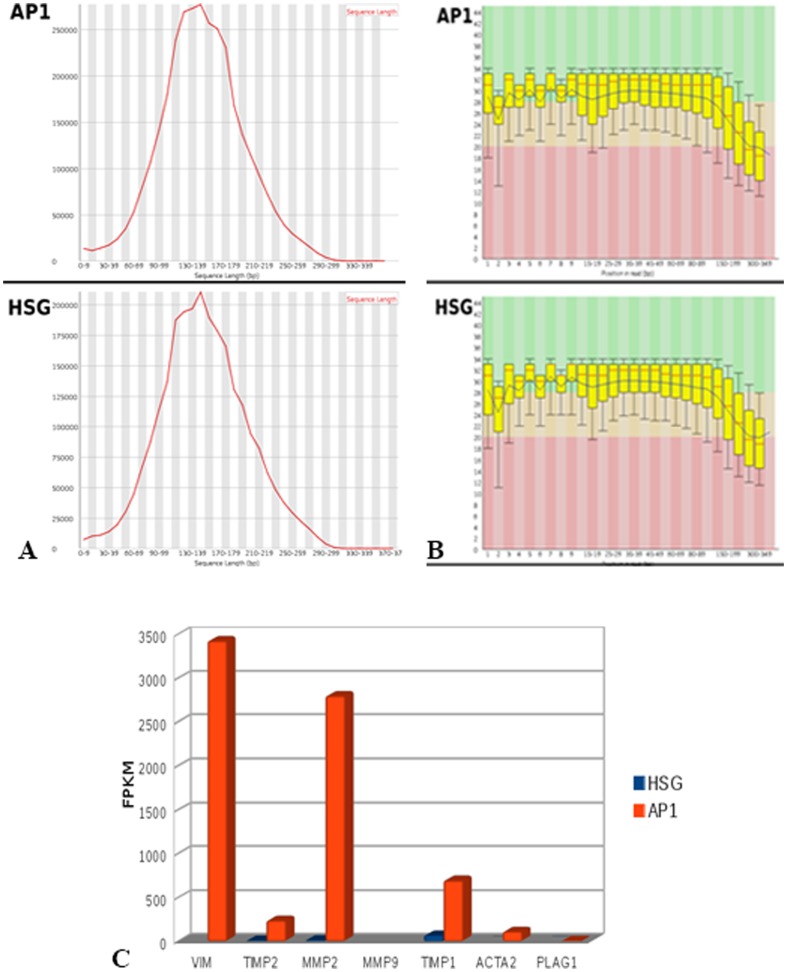
DNA Sequencing. Length distribution of AP-1 and HSG reads. Some reads have short length (6–49 bp), removed to prevent alignment errors (**A**). Quality Graph of AP-1 and HSG libraries. The quality is over Phred 20 in most bases, but for both samples (AP-1 and HSG) the quality decreases in the sequences (**B**). Expression level (Fragments Per Kilobase Of Exon Per Million Fragments Mapped, FPKM) for the genes VIM, TIMP2, MMP2, MMP9,TIMP1, ACTA2 and PLAG1, for AP-1 and HSG library (**C**).

For the AP-1 and HSG libraries, 3.231.730 and 2.522.404 reads were produced, respectively. The graph of length distribution shows a lower frequency of reads shorter than 50 bp (**Fig. 11A**), which were removed to prevent alignment errors or inespecific mapping. Furthermore, the ends of reads with Phred quality bellow 20 were trimmed, despite representing a minimal amount (**Fig. 11B**). After processing this data, 3.231.730 reads remained for AP-1 library, and 2.522.404 reads for HSG.

Both libraries presented a high mapping rate: 3.103.911 reads (98.94%) for AP-1 and 2.286.306 (93.37%) for HSG. Based on this data, the expression of 7 target genes (VIM, TIMP2, MMP2, MMP9, TIMP1, ACTA2 e PLAG1) was evaluated. Only MMP9 was not expressed in both libraries, and VIM was expressed solely in AP-1 library (**Table 2**). The major difference regarding the gene expression level between the AP-1 and HSG samples occurred for MMP2, which was 184 times more expressed in AP-1 cells (**Fig. 11C**).

**Table 2:** Gene expression (Fragments Per Kilobase Of Exon Per Million Fragments Mapped, FPKM) for the target genes in HSG and AP-1 libraries.

Gene	HSG	AP-1
VIM	0	3422,41
TIMP2	9,84674	236,372
MMP2	15,1939	2796,26
MMP9	0	0
TIMP1	67,5746	692,614
ACTA2	0,393327	109,048
PLAG1	0,394537	7,97945

The RNA-Seq data was produced to validate the expression profile observed for genes VIM, TIMP2, MMP2, MMP9, TIMP1, ACTA2 and PLAG1. In addition, the normalized transcript abundance is presented in Table 1 in FPKM (Fragments Per Kilobase Of Exon Per Million Fragments Mapped) column.

## Discussion

A novel cell line named AP-1 has been propagated from human pleomorphic adenoma. These cells expressed myoepithelial markers, proteases and their inhibitors, such as MMP2, MMP9, TIMP1 and TIMP2. These findings were confirmed by transcriptomic analysis. AP-1 cells also displayed numeric and structural chromosomal alterations, which may be directly involved in tumorigenesis.

Salivary gland tumors show variable clinical behavior and morphological features. Special interest has been directed to pleomorphic adenoma, due not only to its frequency, but also to its histopathological diversity [1], [2]. The pleomorphic adenoma sample used in this study consisted in a mixture of spindle and plasmacytoid mioepithelial cells distributed as sheets, cords and islets, embedded in a myxoid/chondroid stroma. This histopathological diversity has been attributed mainly to the presence of myoepithelial cells in different degrees of differentiation [1], [2].

Pleomorphic adenoma is reported to be a great source of myoepithelial cells. Tumor cells *in vivo* showed important myoepithelial markers such as vimentin, S-100 protein, cytokeratin and smooth muscle actin. These markers were also observed in AP-1 cells. In addition, transcriptomic analysis confirmed immunohistochemistry findings. Vimentin and α-smooth muscle actin were more expressed in AP-1 compared to HSG cells (human salivary gland). Cytogenetic analysis showed various numerical abnormalities, which could create gene extra copies. Chromosome 10 contains the *VIM* gene (region 10p12.33) [33], which encodes vimentin. Interestingly actin is encoded by the gene *ACTA2*, also located on chromosome 10 (region 10q23.31) [33].

Furthermore, AP-1 cells were positive for cytokeratin, an epithelial cells marker, corroborating their parenchymal origin. Hence, AP-1 cells are a mixed population of neoplastic epithelial cells and myoepithelial cells, similar to pleomorphic adenoma *in vivo*, maintaining their original phenotypic characteristics.

Our AP-1 cell line showed spindle-shaped cells with well-developed Golgi complex, mitochondria, glycogen granules, and networks of filamentous structures in the cytoplasm near the plasma membrane compatible with some features of PA cell lines [18]-[21]. Growth curve showed that AP-1 cells grew steadily until the 5th day, with a doubling time of 3.342 days. Three-dimensional culture inside Matrigel showed that AP-1 cells recapitulated tumor architecture.

The AP-1 cell line has been cultured for at least 18 passages. In most of the passages, we conducted phenotype characterization by immunofluorescence and electron microscopy. Since the 5^th^ passage, all cells maintained the same markers and no phenotype modifications have been observed. Moreover, no fibroblastic features were found at any time.

Although classified as a benign tumor with slow growth, pleomorphic adenoma can be locally invasive, forming large lesions [34]. Therefore, other mechanisms may be responsible for tumor spreading along the normal surrounding tissue. However, the mechanisms regulating the remodeling and local invasiveness of pleomorphic adenoma remain elusive.

It is well known that MMP-mediated extracellular matrix degradation is a crucial factor in tumor invasion and metastasis [9], [12]. These enzymes have a low physiologic expression, but are found in high levels in many tumors [12]. Immunofluorescence revealed that AP-1 cells were positive for MMP2 and MMP9, and zymography showed secretion of both active and latent forms of MMP2, and latent form of MMP9.

Transcriptomic analysis confirmed our findings. A major gene expression level was found for MMP2, which is 184 times higher in AP-1 cells compared to HSG cells. However, MMP9 gene was not expressed in both libraries, maybe because the sequencing coverage produced by the Ion Torrent PGM is not enough to represent rare transcripts. Quantitative fluorescence also showed that MMP2 staining was clearly higher compared to MMP9. Thus, transcriptomic, immunofluorescence and zymographic analysis were consistent. The MMP2 gene is located on chromosome 16 (16q13-q21) [33]. Karyotype analysis showed that this chromosome presented various numerical abnormalities, which may indicate that extra copies of this gene may be related to an increased expression of MMP2.

Immunofluorescence and transcriptomic analysis showed that TIMP-1 and TIMP-2 were significantly expressed in AP-1 cells. TIMPs 1 and 2 inhibit MMP activity [34], [35]. On the other hand, TIMPs have been related to other cellular functions, such as cell growth, angiogenesis, cancer metastasis, and anti-apoptotic activity [36], [37]. In AP-1 cells, colocalization of MMPs and TIMPs was found. Taken together, our results suggested that MMPs and TIMPs are expressed by AP-1 cells, and may combine forces to regulate invasive events related to this neoplasm.

The genetic events involved in pleomorphic adenoma progression are not entirely understood. Marked numerical chromosomal aberrations were found in AP-1 cell line (60% polyploidy) and this change was also described for other PA cell lines [21]. Conventional cytogenetic analysis has detected recurrent translocations in approximately 70% of pleomorphic adenomas [38], [39]. These translocations target primarily PLAG1 (pleomorphic adenoma gene 1), resulting in upregulation of this gene, located at 8q12 [40], [41]. Based on the current state of knowledge, translocations involving PLAG1 are specific for two pathologic entities, pleomorphic adenoma and lipoblastoma [42], [43]. Transcriptomic study showed a high expression of PLAG1 in AP-1 cells compared to HSG cell line.

Overall, the cell line derived from human pleomorphic adenoma (AP-1 cells) was successfully established and characterized. Pleomorphic adenoma *in vivo* local invasiveness may be regulated by MMPs and TIMPs. The significance of chromosomal alterations in AP-1 cells remains to be elucidated.
